# Comparative genomic hybridization and histological variation in primitive neuroectodermal tumours

**DOI:** 10.1038/sj.bjc.6690525

**Published:** 1999-07

**Authors:** J C Nicholson, F M Ross, J A Kohler, D W Ellison

**Affiliations:** Wessex Regional Genetics Laboratory, Salisbury, Wiltshire, SP2 8BJ, UK; Departments of Child Health and, Pathology, Southampton General Hospital, Southampton, SO16 6YD, UK

**Keywords:** medulloblastoma, cerebral PNET, comparative genomic hybridization, isochromosome 17q, monosomy 22, glial fibrillary acidic protein

## Abstract

The objective of this study was to test the hypothesis that chromosomal imbalances in central nervous system primitive neuroectodermal tumours (PNETs) reflect site and histology. We used comparative genomic hybridization to study 37 cases of PNET, of which four were cerebral and 31 were medulloblastomas classified histologically as classic (*n* = 17) or nodular/desmoplastic (*n* = 14). Tumour immunophenotype was characterized with antibodies to neuroglial, mesenchymal and epithelial markers. Chromosomal imbalances were detected in 28 medulloblastomas (90%), and significant associations between tumour variants and genetic abnormalities were demonstrated. Aberrations suggesting isochromosome 17q were present in eight (26%) medulloblastomas, of which seven were classic variants. None of these cases, or a further six with gain of 17q, showed immunoreactivity for glial fibrillary acidic protein. Loss on 9q was found in six cases (19%), five of them nodular/desmoplastic. Loss of 22 occurred in four (13%), all classic medulloblastomas in young patients with a poor outcome and immunoreactivity for more than one epithelial or mesenchymal marker. Different patterns of imbalance were found in the cerebral PNETs. There were no abnormalities of chromosome 17, but all three cases with imbalance showed losses of 3p12.3–p14. © 1999 Cancer Research Campaign


*British Journal of Cancer* (1999) **80**(9), 1322–1331
© 1999 Cancer Research Campaign
Article no. bjoc.1999.0525

### Comparative genomic hybridization and histological variation in primitive neuroectodermal tumours

**JC Nicholson[1,2], FM Ross[1], JA Kohler[2] and DW Ellison[3]**

[1]Wessex Regional Genetics Laboratory, Salisbury, Wiltshire SP2 8BJ, UK; Departments of [2]Child Health and [3]Pathology, Southampton General Hospital, Southampton SO16 6YD, UK

**Summary** The objective of this study was to test the hypothesis that chromosomal imbalances in central nervous system primitive neuroectodermal tumours (PNETs) reflect site and histology. We used comparative genomic hybridization to study 37 cases of PNET, of which four were cerebral and 31 were medulloblastomas classified histologically as classic ($n = 17$) or nodular/desmoplastic ($n = 14$). Tumour immunophenotype was characterized with antibodies to neuroglial, mesenchymal and epithelial markers. Chromosomal imbalances were detected in 28 medulloblastomas (90%), and significant associations between tumour variants and genetic abnormalities were demonstrated. Aberrations suggesting isochromosome 17q were present in eight (26%) medulloblastomas, of which seven were classic variants. None of these cases, or a further six with gain of 17q, showed immunoreactivity for glial fibrillary acidic protein. Loss on 9q was found in six cases (19%), five of them nodular/desmoplastic. Loss of 22 occurred in four (13%), all classic medulloblastomas in young patients with a poor outcome and immunoreactivity for more than one epithelial or mesenchymal marker. Different patterns of imbalance were found in the cerebral PNETs. There were no abnormalities of chromosome 17, but all three cases with imbalance showed losses of 3p12.3–p14.

**Keywords:** medulloblastoma; cerebral PNET; comparative genomic hybridization; isochromosome 17q; monosomy 22; glial fibrillary acidic protein

Tumours of the central nervous system (CNS) are the commonest solid neoplasms of childhood, and primitive neuroectodermal tumours (PNETs) are the most numerous among these, accounting for approximately 25% of all childhood brain tumours (Packer, 1995). More than 90% of cases of CNS PNET occur in the posterior fossa as medulloblastomas. PNETs at other CNS sites resemble the classic medulloblastoma, which has a propensity for divergent differentiation along neuro-epithelial lines, manifesting as expression of glial fibrillary acidic protein (GFAP) and/or neuronal proteins or rarely as the presence of scattered ganglion cells (Tomlinson et al, 1992; Ellison and Love, 1998). In addition to the classic medulloblastoma, several variants are recognized in the World Health Organization classification of CNS tumours: desmoplastic medulloblastoma, melanotic medulloblastoma and medullomyoblastoma (Kleihues et al, 1993). Desmoplastic medulloblastomas account for approximately 20% of medulloblastomas and frequently exhibit a nodular architecture in which cells often show a neuronal or glial immunophenotype; these nodular/desmoplastic medulloblastomas are considered to form a histological spectrum of tumours, distinct from classic medulloblastomas (Giangaspero et al, 1991; Tomlinson et al, 1992).

Cytogenetic studies of medulloblastoma and other PNETs have identified several non-random chromosomal aberrations in a high proportion of cases (Biegel et al, 1989; Bhattachajee et al, 1997; Bigner et al, 1997). Of these, isochromosome 17q [i(17q)] is the most common, occurring in a third of cases overall (Mertens et al, 1994). Other recognized patterns of abnormality have included loss of the X or Y chromosome, trisomy 7, monosomy 22, double minutes and structural rearrangements, particularly of chromosomes 1, 7 and 11 (Heim and Mitelman, 1995; Bigner et al, 1997). Microsatellite analysis has corroborated the existence of chromosome 17 abnormalities by documenting loss of heterozygosity (LOH) on 17p, as well as on chromosomes 9, 10, 11, 16 and 22 (Blaeker et al, 1996).

All of these abnormalities result in genetic imbalance and therefore make these tumours particularly suitable for study by comparative genomic hybridization (CGH). Two previous studies (Schütz et al, 1996; Reardon et al, 1997) found widely different numbers of abnormalities in medulloblastomas using CGH. The aims of this study were to screen a series of CNS PNETs, mostly medulloblastomas, for chromosomal imbalance as revealed by CGH, to test the hypothesis that histological and immunophenotypical differences among variants of PNET are associated with specific genetic abnormalities, and to assess the prognostic value of any commonly observed genetic abnormalities.

### MATERIALS AND METHODS

### Patient group

All biopsies ($n = 39$) came from patients ($n = 37$) attending the Wessex Neurological Centre in Southampton between 1985 and 1997. Some tumour, surplus to diagnostic requirements, was stored in each case at –170°C. All specimens used in the study were obtained prior to commencement of treatment, except in cases 8 and 13.

Most patients ($n = 31$) had a medulloblastoma. Of these, 17 showed classic histology, while the remaining 14 had a

*Received 16 November 1998*
*Revised 21 January 1999*
*Accepted 27 January 1999*

*Correspondence to:* JC Nicholson, Department of Child Health, Mail Point 0021, G Level, Southampton General Hospital, Tremona Road, Southampton SO16 6YD, UK

**Table 1** Clinical features of PNETs and CGH results

| Case | Age at diagnosis (years) | PNET variant | Metastases | Primary surgical resection | Chemotherapy | Radiotherapy | Survival (years) | CGH results |
|---|---|---|---|---|---|---|---|---|
| 1 | 19.8 | Classic MB | No | Subtotal | No | CS | 4.8* | rev ish enh(5,6,7,12q24,17,18),dim (X,2q14qter,3,8p12q24.1,9p,11,13q13q31, 14q23q31,16q) |
| 2 | 14.3 | Classic MB | No | Partial | Yes | CS | 8.8* | rev ish enh(7,17q),dim(2,8,11p, 12pterq22,13q,17p) |
| 3 | 8.5 | Classic MB | No | Subtotal | Yes | CS | 3.3 | rev ish enh(4p15.3pter,6q23qter, 9p,12q23qter,17q),dim(Y,5q34qter,8p12pter, 17p,18p,20) |
| 4 | 0.7 | Classic MB | No | Partial | No | No | 0.3 | rev ish dim(22) |
| 5 | 1.6 | Classic MB | No | Partial | Yes | No | 0.3 | rev ish enh(17,19q), dim(Yq,11q13.5q23) |
| 6 | 6.3 | Classic MB | Spinal | Complete | Yes | CS | 1.4 | rev ish enh(17q,18),dim(8,17p) |
| 7 | 9.8 | Classic MB | Spinal | Partial | Yes | CS | 1.7 | rev ish enh(17q),dim(Yq,17p) |
| 8 | 6.5 | Classic MB | No | Subtotal | Yes | CS | 5.1 | 1st relapse: rev ish enh(1q,4p16,7q, 12q23qter,17q21qter),dim(4q22q28, 4q32qter, 5q33qter,8p21.3pter, 11p11.2p13,16q21qter) 2nd relapse: rev ish enh(1q,4p16,7q, 12q23qter, 17q21qter),dim(Y,4q22q25, 4q32qter, 5q33qter,8p21.3pter,11p, 16q21qter) |
| 9 | 6.6 | Classic MB | No | Complete | Yes | CS | 2.8* | No imbalances |
| 10 | 3.3 | Classic MB | No | Complete | No | CS | 6.3* | rev ish enh(1q,8,17q),dim(4q31qter,17p) |
| 11 | 24.8 | Classic MB | Spinal | Subtotal | No | CS | 0.3* | rev ish enh(10,17q),dim(17p) |
| 12 | 4.8 | Classic MB | Spinal | Complete | No | No | 0.1 | rev ish enh(2p13.3pter,12,17q,18), dim(4q24qter,16q21qter,17p) |
| 13 | 30.3 | Classic MB | No | Subtotal | No | CS | 3.3 | 1st relapse: rev ish enh(Xq,3q,6q26q27,9p, 13,15q23qter,18p),dim(Xp,4q31q34, 9q22q34.1,10q21qter,11p14,11q14q22, 14,15q11q15,17p12pter),amp(2q14q21) |
| 14 | 32.2 | Classic MB | Not known | Biopsy | No | No | 0 | rev ish dim(10q,11,17p) |
| 15 | 5.3 | Classic MB | No | Subtotal | Yes | CS | 1.5 | rev ish dim(22) |
| 16 | 0.3 | Classic MB | No | Biopsy | No | No | 0 | rev ish dim(22) |
| 17 | 0.7 | Classic MB | Spinal | Subtotal | Yes | Local | 1.3 | rev ish enh(14),dim(22) |
| 18 | 9.3 | ndMB | Spinal | Subtotal | Yes | CS | 0.8 | no imbalances |
| 19 | 5.1 | ndMB | No | Subtotal | Yes | CS | 6.4* | rev ish enh(4,5,6,7,17q),dim(Y,8,17p) |
| 20 | 11.2 | ndMB | No | Subtotal | No | CS | 1.3* | rev ish enh(1,7,17q),dim(Xq12q25,Y,8,10,11, 13q13q21,14q11.2q13,14q24,15,20) |
| 21 | 5.9 | ndMB | No | Subtotal | Yes | CS | 1.2* | rev ish enh(2,7,15,17q),dim(3,8,10, 11p15pter,21) |
| 22 | 5.3 | ndMB | No | Subtotal | No | No | 0.1 | rev ish enh(7q,8q24,12q24,17q22qter,18, 19pterq12),dim(8p,10q21.3qter,19q13.2qter) |
| 23 | 12.7 | ndMB | No | Complete | Yes | CS | 8.1* | rev ish enh(1q31qter,2,4q21q25,9p,18), dim(4p15.2q13,4q26q32,8,9q22q34,10,16), amp(12p13,13q33) |
| 24 | 3.6 | ndMB | No | Complete | No | CS | 7.4* | rev ish dim(9q21q31) |
| 25 | 19.6 | ndMB | No | Subtotal | No | CS | 4.5 | Primary tumour: rev ish enh(3q),dim(Y) 1st relapse: rev ish enh(3q),dim(11q13q22) |
| 26 | 22.8 | ndMB | No | Complete | No | CS | 7.2* | rev ish dim(X,6q16q24) |
| 27 | 7.3 | ndMB | No | Complete | No | CS | 1.3* | rev ish enh(1q,2,3p14p21,7,9p,10p,13), dim(Xp,3q,4pterq26,5q,8q12qter,9q21.3q22, 10q,11q23qter,14,16,17p,18p),amp(4q28) |
| 28 | 30.3 | ndMB | No | Subtotal | No | CS | 2.4 | rev ish enh(20q),dim(9q22qter,17p) |
| 29 | 25.6 | ndMB | Not known | Complete | No | Local | 6.4 | rev ish enh(15q12q14) |
| 30 | 27.9 | ndMB | Bone | Partial | No | CS | 5.6 | No imbalances |
| 31 | 4 | ndMB | No | Subtotal | No | CS | 0.9* | rev ish enh(3,9p),dim(9q) |
| 32 | 11.9 | GNB | No | Complete | No | CS | 10.3* | rev ish enh(17q),dim(17p) |
| 33 | 14.1 | MmyoB | Not known | Complete | No | CS | 10.3* | rev ish enh(8) |
| 34 | 8.3 | cPNET | No | Biopsy | Yes | Palliative | 0.6 | rev ish enh(X,1,7p,9,13,16p,19,20,22),dim (3,4,5,6,8q13qter,10q11q22,10q24.3qter, 12p12q23,14,15q11q23,16q12q22.1,18q), amp(7p11.2p12,7q21.3q22) |
| 35 | 2.6 | cPNET | No | Subtotal | Yes | CS | 5.7* | rev ish enh(X,1p32pter,1q32qter,2p, 7q33qter),dim(3p12p14,3p21.3pter,5pterq33, 6,7p12p21.1,9p23q32,11,14q24qter) |
| 36 | 6.4 | cPNET | Not known | Partial | No | No | 0.2 | rev ish dim(3p13p21.1) |
| 37 | 1.1 | cPNET | No | Complete | Yes | No | 0.8 | No imbalances |

Survival: *denotes ongoing remission. MB, medulloblastoma; nd, nodular/desmoplastic; GNB, ganglioneuroblastoma; MmyoB, medullomyoblastoma; cPNET, cerebral PNET; CS, craniospinal radiotherapy.

*© 1999 Cancer Research Campaign*

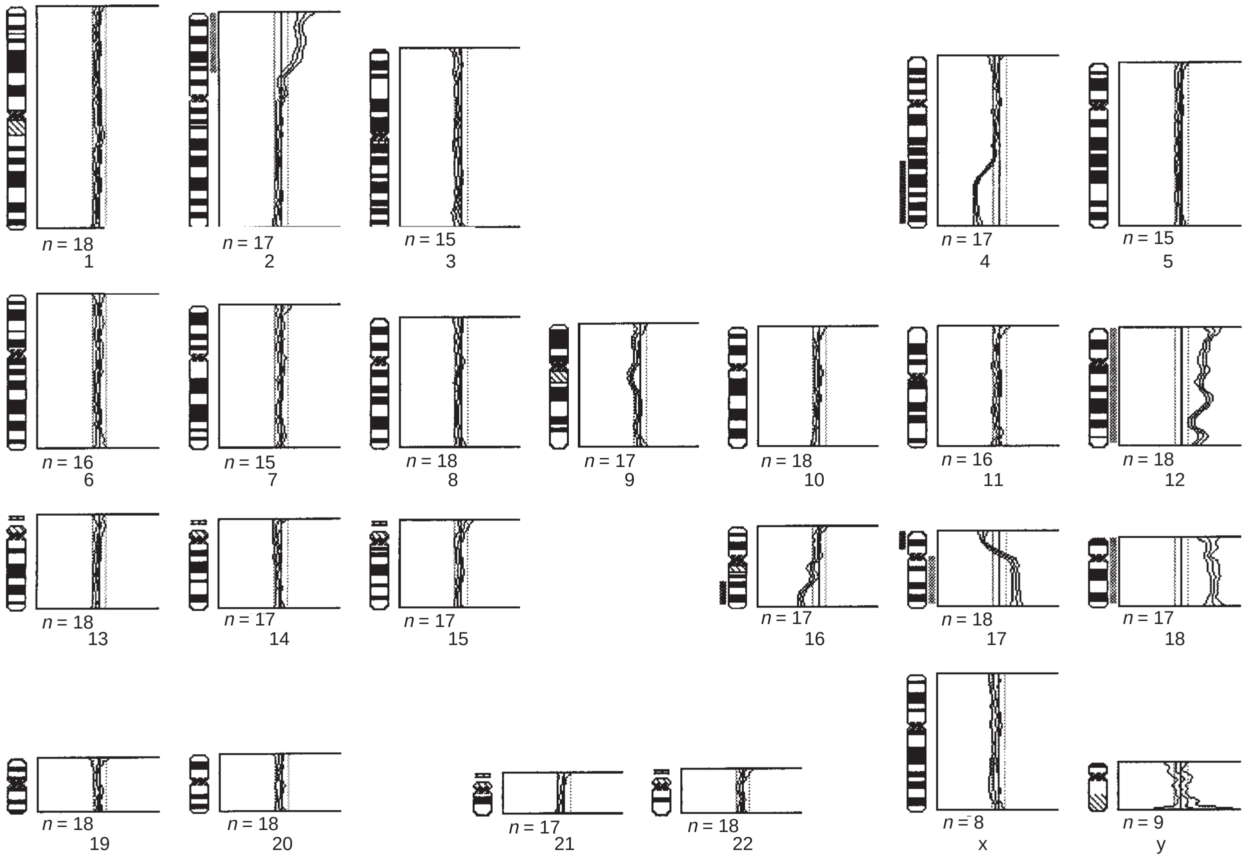

**Figure 1** CGH ratio profiles for case 12. For each chromosome profiled, the three vertical lines correspond to green:red fluorescence intensity ratios of 0.9, 1.0 and 1.1. Each profile is expressed as a mean with 99% confidence intervals (CIs), and the number of chromosomes analysed in each case is shown beneath the profile. Significant imbalances are represented by solid bars to the left or right of the chromosome ideograms, for losses or gains respectively, excluding heterochromatic regions nodular/desmoplastic architecture. For two of the classic medulloblastomas, material was only available for analysis from recurrent tumour. In one nodular/desmoplastic case, material was available from biopsies both at diagnosis and relapse. A medullomyoblastoma, a posterior fossa ganglioneuroblastoma and four cerebral PNETs comprised the remaining six tumours.

Clinical details are provided in Table 1. The median age at surgery of patients with medulloblastoma was 7.3 years (range 0.3–32.2 years). The male:female ratio was 5.2:1. Spinal cord deposits were present at diagnosis in six patients, and one patient presented with bone metastases. A complete macroscopic surgical removal was possible with nine medulloblastomas. Adjuvant therapy was given to 26 patients; 13 patients received chemotherapy to their primary tumour and 25 radiotherapy, which was localized to the tumour bed in two cases and to the craniospinal axis in the remainder. The six patients who received no radiotherapy died within 4 months of diagnosis. Of the remaining patients with medulloblastoma, 13 were alive at last review and all were still in remission, between 4 and 109 months from diagnosis.

The small group of cerebral PNETs had a median age at diagnosis of 4.5 years (range 1.1–8.3 years). One patient is alive and disease-free after 68 months, following both chemotherapy and craniospinal radiotherapy, and the remaining patients died between 2 and 9 months after diagnosis, two of them having received chemotherapy and one also palliative radiotherapy.

### Histochemistry

Near adjacent sections (5μm) were cut from formalin-fixed, paraffin-embedded tissue. A reticulin preparation (Gordon and Sweet) was used to aid assessment of desmoplasia and nodular architecture. Immunohistochemistry was undertaken as previously described (Ellison et al, 1995) using antibodies to the neuroepithelial proteins GFAP (Pathology Department, University of Southampton), synaptophysin (DAKO) and neurofilament protein (DAKO), and to the non-neuroepithelial proteins epithelial membrane antigen (DAKO), desmin (Europath), smooth muscle actin (Sigma) and low molecular weight cytokeratins (Becton Dickinson). Histological assessment was undertaken without knowledge of the results of CGH.

### Comparative genomic hybridization

CGH was performed on frozen tumour tissue using a protocol modified from that of Kallioniemi and co-workers (1994). A section of this tissue was prepared for histology to ascertain the degree of contamination with non-neoplastic tissue. Of 39 tumour specimens, 30 consisted solely of neoplastic cells while the proportion of non-neoplastic tissue in the remainder ranged from approximately 5–30%. Tissue (5–30 mg) was digested in a solution of proteinase K at 37°C. DNA was obtained by salt extraction (Miller

 *© 1999 Cancer Research Campaign*

**Table 2** Immunophenotype of PNETs

| | Tumour | GFAP | SYN | NFP | CK | EMA | SMA | DES |
|---|---|---|---|---|---|---|---|---|
| 1 | MB | – | – | – | – | – | – | – |
| 2 | MB | – | – | – | – | – | – | – |
| 3 | MB | – | – | – | – | – | – | – |
| 4 | MB | – | – | – | – | 2+ | 2+ | – |
| 5 | MB | – | 1+ | – | – | – | – | – |
| 6 | MB | – | 1+ | – | it | it | it | it |
| 7 | MB | – | 1+ | – | it | it | it | it |
| 8a | MB | – | it | it | it | it | it | it |
| 8b | MB | – | 2+ | – | – | – | – | – |
| 9 | MB | – | 2+ | – | – | – | – | – |
| 10 | MB | – | 2+ | 3+ | – | – | – | – |
| 11 | MB | – | 3+ | – | – | – | – | – |
| 12 | MB | – | 3+ | – | – | – | – | – |
| 13 | MB | 1+ | – | – | – | – | – | – |
| 14 | MB | 1+ | – | – | – | – | 2+ | – |
| 15 | MB | 1+ | – | – | – | 3+ | 3+ | – |
| 16 | MB | 1+ | – | – | 2+ | 3+ | 2+ | – |
| 17 | MB | 2+ | – | – | 3+ | 3+ | – | – |
| 18 | nd MB | – | 2+ | – | – | – | – | – |
| 19 | nd MB | – | 3+ | – | – | – | – | – |
| 20 | nd MB | – | 3+ | – | – | – | – | – |
| 21 | nd MB | – | 3+ | – | – | – | – | – |
| 22 | nd MB | – | 3+ | – | – | – | – | – |
| 23 | nd MB | 1+ | – | – | – | – | – | – |
| 24 | nd MB | 2+ | – | – | – | – | – | – |
| 25a | nd MB | 2+ | – | – | – | – | 2+ | – |
| 25b | nd MB | 2+ | – | – | – | – | 1+ | – |
| 26 | nd MB | 2+ | – | – | it | it | it | it |
| 27 | nd MB | 2+ | 1+ | – | – | – | 2+ | – |
| 28 | nd MB | 2+ | 2+ | – | – | – | – | – |
| 29 | nd MB | 2+ | 2+ | – | it | it | it | it |
| 30 | nd MB | 2+ | 3+ | 3+ | it | it | it | it |
| 31 | nd MB | 3+ | – | – | – | – | – | – |
| 32 | GNB | – | 1+ | 1+ | – | – | – | – |
| 33 | MmyoB | – | 1+ | – | – | – | – | 2+ |
| 34 | cPNET | – | – | – | – | – | – | – |
| 35 | cPNET | – | – | – | – | it | – | – |
| 36 | cPNET | – | – | – | it | it | – | – |
| 37 | cPNET | 1+ | – | – | it | it | it | it |

PNETs contained either no labelled cells (–), a small number of scattered immunoreactive cells (1+), less than 50% of cells with immunoreactivity (2+), or more than 50% of cells with immunoreactivity (3+). GFAP: glial fibrillary acidic protein; SYN: synaptophysin; NFP: neurofilament protein; CK: low molecular weight cytokeratins; EMA: epithelial membrane antigen; SMA: smooth muscle $\alpha$ actin; DES: desmin; nd = nodular/desmoplastic; it = insufficient tissue.

et al, 1988) and directly labelled by nick translation with fluorescein-12-dUTP (Dupont NEN). Reference DNA was extracted from blood of karyotypically normal individuals and labelled with Texas red-5-dUTP (Dupont NEN). Approximately 650 ng of labelled DNA fragments in the size range 300–3000 base pairs were hybridized with 30 µg human Cot-1 DNA (GibcoBRL) for 3 days at 37°C on to normal male target metaphase slides (Vysis). Following hybridization, the slides were washed, air-dried and mounted in anti-fade solution containing 1.5 µg $ml^{-1}$ 4,6′-diamino-2-phenylindole counterstain (Vector Laboratories). A control slide with co-hybridized labelled DNA from a normal male and female was included with each experiment.

### Digital image analysis

Slides were analysed using a Zeiss Axioskop fluorescence microscope and images captured by a cooled charged couple device camera (Photometrics) in conjunction with Smartcapture software (Digital Scientific), and then enhanced and analysed using Quips CGH software (Vysis). Each metaphase used in the analysis was karyotyped and green to red fluorescence intensity ratios along the length of each chromosome were calculated. Data from 5–10 metaphases were combined to give a mean ratio profile for each chromosome together with profiles corresponding to 99% confidence intervals (CIS), as calculated by the Quips software. Gains or losses of material by the tumour were deduced from deviations of the mean profile beyond thresholds set at ratios of 1.1 and 0.9, provided also that the 99% CIs consistently deviated to the same side of the midline for the regions involved, and that no deviations were seen in the corresponding regions in the normal control performed with each experiment. Amplifications were directly visualized by microscopy as discrete regions of gain and were localized by ratios greater than 1.5:1. Apparent gains or losses in heterochromatic regions were excluded from analysis.

The abnormalities identified by CGH were expressed according to the International System for Human Cytogenetic Nomenclature (ISCN, 1995). CGH analysis was carried out without knowledge of histology results.

© 1999 Cancer Research Campaign 

### Statistical analysis

Survival analyses were undertaken for medulloblastomas using Kaplan–Meier estimates and correlations were assessed in contingency tables. Survival time was calculated in months from craniotomy to death, but in surviving (censored) patients, the interval between operation and last medical review was used in the analysis.

### RESULTS

### CGH analysis

Chromosomal imbalances were detected by CGH in all but four tumours, and most cases showed multiple abnormalities, with a mean of 6.2 per tumour (Table 1). The profiles for case 12 are illustrated in Figure 1. The imbalances in the 31 classic and nodular/desmoplastic medulloblastomas are shown in Table 1 and in Figure 2. In our control experiments, using co-hybridized reference DNA from a normal male and female, no imbalances reaching our criteria for inclusion were detected on the autosomes, including the CG-rich regions 1p, 16p, 19 and 22.

Chromosome 17 was most frequently affected in medulloblastomas, showing abnormalities in 18 tumours (58%). Twelve cases (39%) showed loss of 17p, of which eight (26%) had a reciprocal gain of 17q, suggesting the presence of i(17q). Four cases had gains of 17q without loss of 17p and two had gains of the whole of 17, making a total of 14 (45%) with gains of all or part of chromosome 17. Other chromosomes frequently affected by losses were 8 (11 cases; 35%), 11 (ten cases; 32%), Y (seven cases; 27% of males), 4 (seven cases; 23%), 9 (seven cases; 23%) and 10q (seven cases; 23%). Common sites of chromosomal gain were 7 (eight cases; 26%), 18 (six cases; 19%), 1q, 9p and 12q (five cases each; 16%). Extensive loss of chromosome 22, suggesting monosomy 22, was seen in four cases. The eight cases with gain of all of 7 or 7q also showed losses of all or part of chromosome 8. Of ten cases with losses on 8p, eight also had gains of 17q. High level gains, suggesting gene amplification, were seen in only three tumours of which one (case 23) had two amplicons. The chromosomal regions involved in amplification were 2q14–q21 (case 13), 4q28 (case 27), 12p13 and 13q33 (case 23). All three cases were associated with multiple regions of imbalance, including losses on 9q, 10q and 16q. In the two tumours for which results were available from two separate biopsies, there were only minor differences between the CGH findings.

The results of CGH analysis for the cerebral PNETs differed from those for the medulloblastomas (Table 1, Figure 3). Both cases 34 and 35 had multiple regions of loss and gain, several of them similar, although case 34 also had two amplicons that mapped to 7p11.2 and 7q21.3. Case 36 had an isolated loss of part of 3p, and case 37 showed no abnormalities, suggesting a balanced karyotype. Of note were the presence of losses involving proximal 3p in all three cases with abnormal CGH findings and the absence of any imbalance involving chromosome 17 in any of the cerebral PNETs studied.

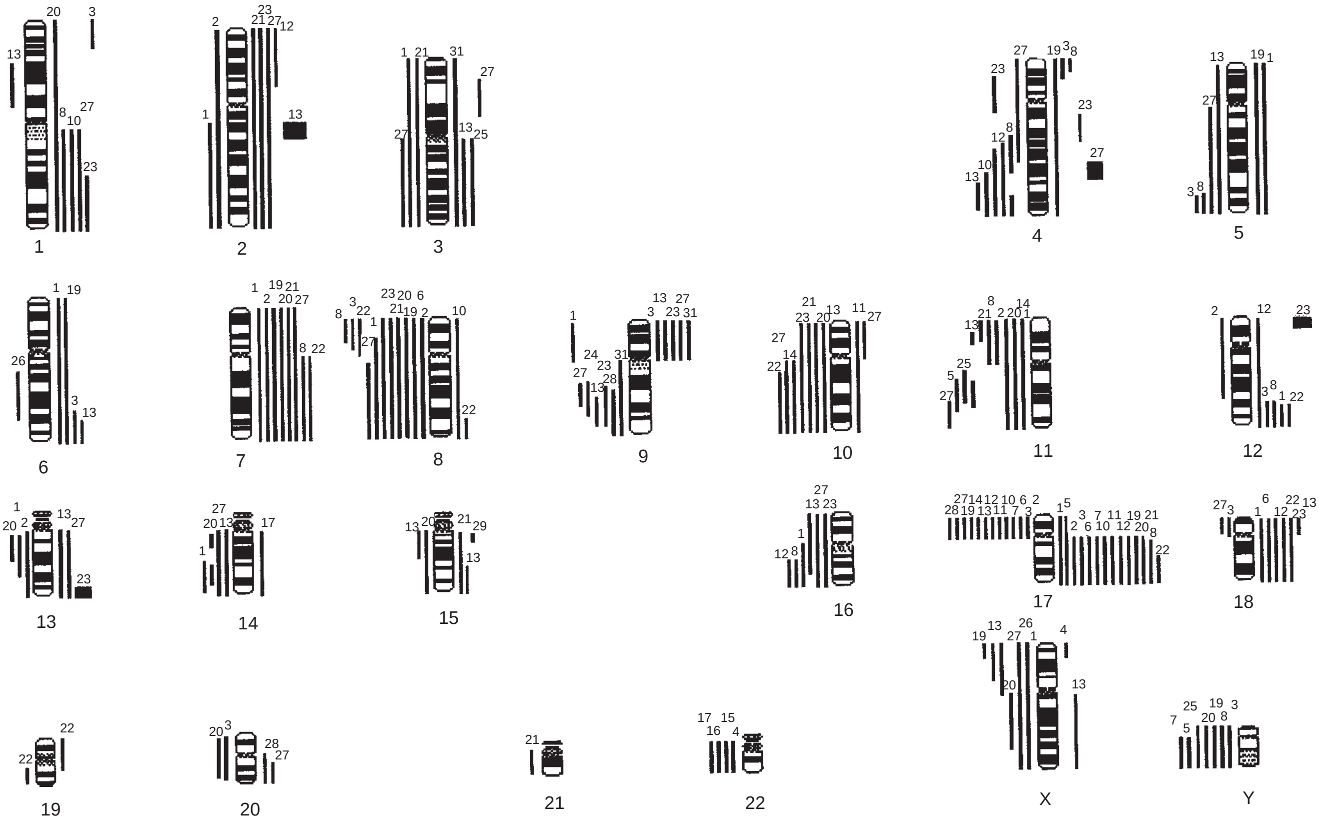

**Figure 2** Summary of regions of chromosomal imbalance identified by CGH in cases of classic and nodular/desmoplastic medulloblastoma. Losses are represented by vertical lines to the left of the chromosome, gains by vertical lines to the right, and amplicons by solid blocks. The cases to which each region of imbalance corresponds are identified by number. Imbalances in cases for which samples from two biopsies were analysed are only included once

 *© 1999 Cancer Research Campaign*

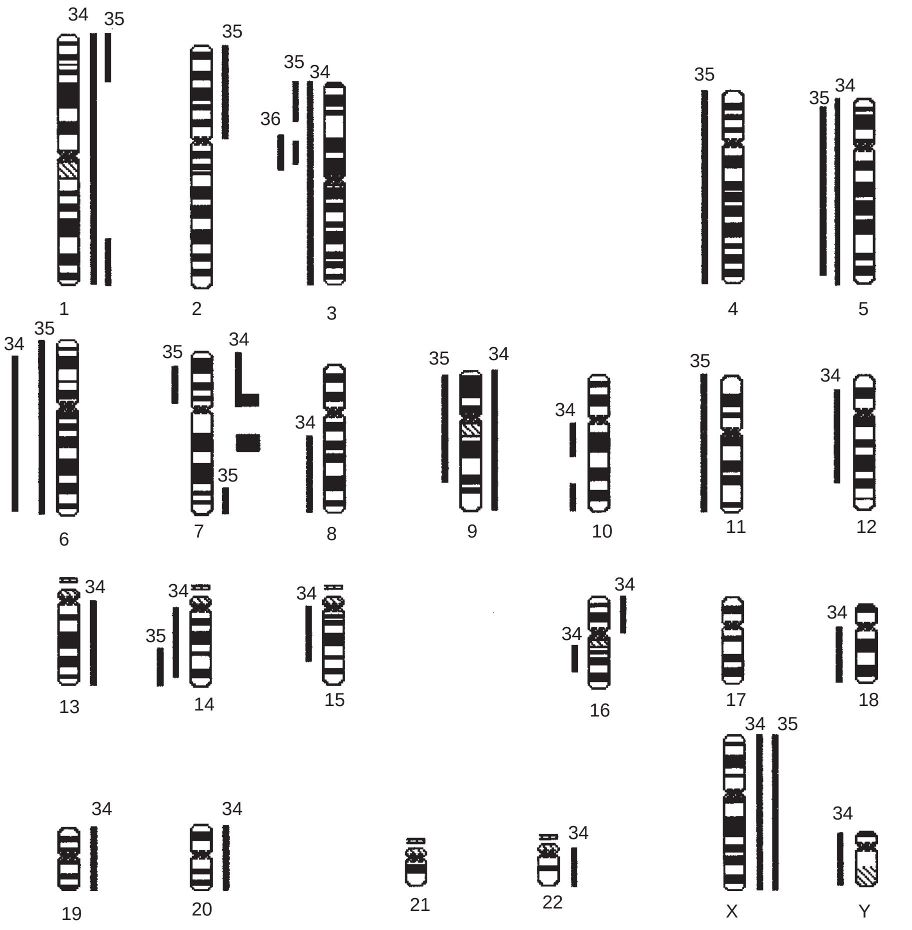

**Figure 3** Summary of regions of chromosomal imbalance identified by CGH in cases of cerebral PNET. Losses are represented by vertical lines to the left of the chromosome, gains by vertical lines to the right, and amplicons by solid blocks. The cases to which each region of imbalance corresponds are identified by number

### Immunohistochemistry

Most medulloblastomas (27/31) were immunoreactive for one or more of the neuroepithelial proteins, GFAP, synaptophysin and neurofilament protein (Table 2). Among classic medulloblastomas, 5/17 were positive for GFAP and 8/16 for either of the neuronal markers synaptophysin and neurofilament protein. In contrast, nodular/desmoplastic medulloblastomas showed a more differentiated immunophenotype; 9/14 were immunoreactive for GFAP and 9/14 for neuronal proteins. An immunophenotype characterized by the presence of more than one non-neuroepithelial protein, including epithelial membrane antigen, was seen in four classic medulloblastomas. Four other medulloblastomas contained tumour cells that labelled with an antibody to smooth muscle actin. These tumours were all GFAP-positive. The medullomyoblastoma contained large desmin-positive cells.

### Chromosomal imbalance and tumour histology

Significant associations between chromosomal imbalance and medulloblastoma histology were sought in contingency tables (Table 3). Imbalance suggesting i(17q) occurred almost exclusively in classic medulloblastomas. Of five tumours with gain of 12q24, four were classic medulloblastomas. In contrast, five out of six tumours with losses on 9q were nodular/desmoplastic medulloblastomas. All tumours with monosomy 22 showed classic medulloblastoma histology.

The same imbalances showed a striking association with the presence or absence of GFAP immunoreactivity (Table 3). For example, all tumours with i(17q) ($n = 8$) were immunonegative for GFAP, as were those showing gain of 17q without reciprocal loss of 17p ($n = 6$). However, the cases with loss of 17p without reciprocal 17q gain ($n = 4$) were GFAP-positive. No significant relationships were found between the results of CGH and immunohistochemistry with antibodies to neuronal proteins. All classic medulloblastomas with monosomy 22 had an immunophenotype characterized by the expression of more than one non-neuroepithelial protein.

### Survival analysis

Kaplan–Meier survival curves showed that patients with evidence of metastases at diagnosis had a significantly poorer prognosis ($P = 0.023$) than those with localized tumours, and that there was a trend ($P = 0.067$) towards longer survival for patients with nodular/desmoplastic rather than classic histology (Figure 4A). The presence of GFAP immunoreactivity had no prognostic value

**Table 3** Abnormalities detected in medulloblastomas by CGH; associations with histological variant and immunoreactivity for GFAP

| MB variant | i(17q) | loss 17p | gain 17q | gain 12q24 | loss 9q22 |
|---|---|---|---|---|---|
| Classic MB | 7 | 9 | 10 | 4 | 1 |
| Nodular/desmoplastic MB | 1 | 3 | 4 | 1 | 5 |
| | $P = 0.031$ | $P = 0.073$ | $P = 0.092$ | $P = 0.217$ | $P = 0.036$ |
| Immunophenotype | i(17q) | loss 17p | gain 17q | gain 12q24 | loss 9q22 |
| GFAP-negative | 8 | 8 | 14 | 5 | 0 |
| GFAP-positive | 0 | 4 | 0 | 0 | 6 |
| | $P = 0.003$ | $P = 0.293$ | $P < 0.0001$ | $P = 0.027$ | $P = 0.003$ |

Figures = number of tumours, Tumours ($n = 8$) with i(17q) are included in columns enumerating tumours with loss of 17p ($n = 12$) and gain of 17q ($n = 14$). MB, medulloblastoma. *P*-values refer to correlation in contingency ($\chi^2$) tables.

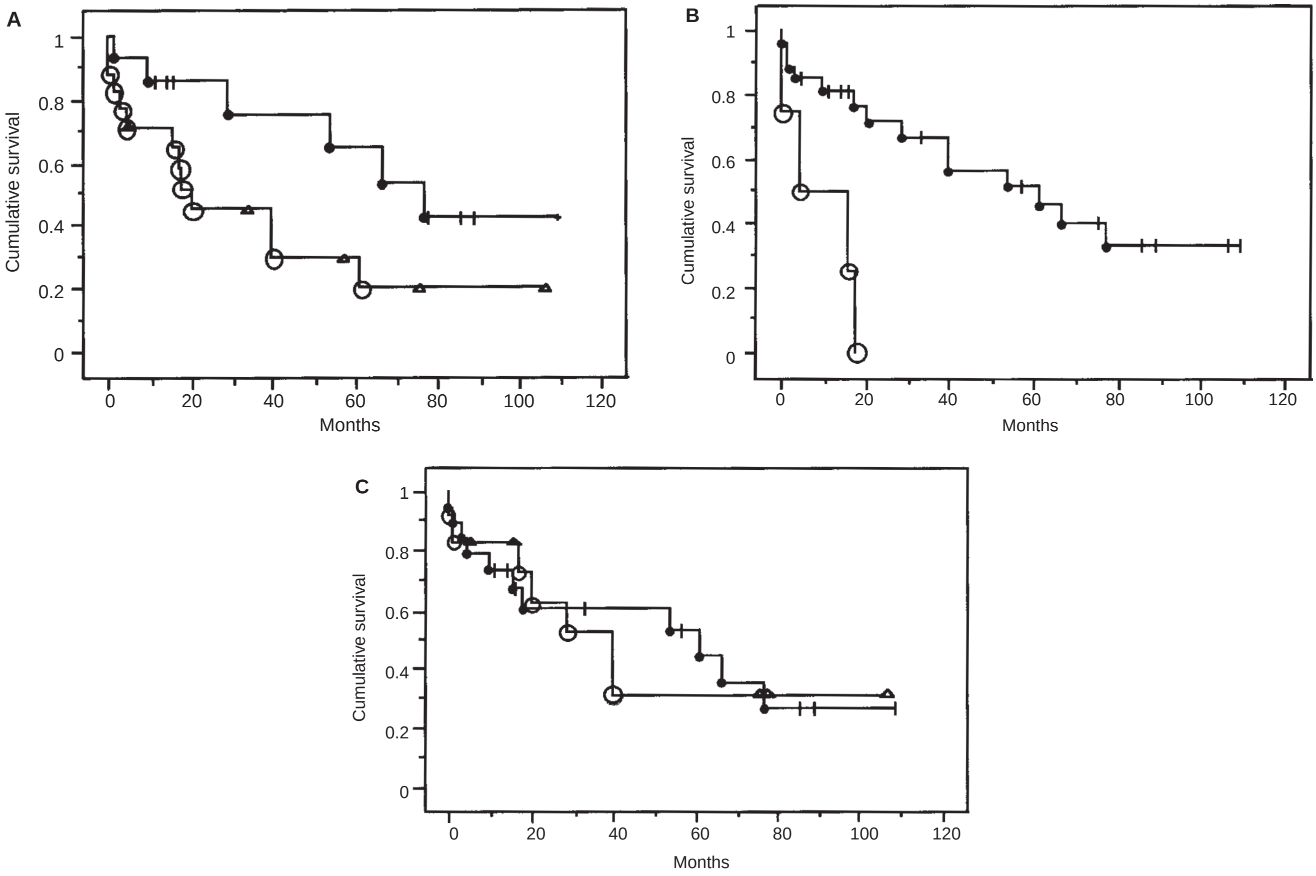

**Figure 4** Kaplan–Meier survival analyses for medulloblastomas, subdivided according to histological subtype (**A**) and the presence or absence of imbalances suggesting monosomy 22 (**B**) or loss of 17p (**C**). Surviving (censored) patients are indicated by bars on the upper curves and, where applicable, by triangles on the lower curves ***A** Classic [○] and nodular/desmoplastic [●] histology ($P = 0.067$); **B** Tumours with [○] and without [●] loss of 22 ($P = 0.0013$); **C** Tumours with [○] and without [●] loss of 17p ($P = 0.94$)

($P = 0.96$). Significantly different survival curves were demonstrated for patients whose tumours had monosomy 22 ($P = 0.0013$), but not for those with loss of 17p ($P = 0.94$) (Figure 4B,C), or any other genetic abnormality.

### DISCUSSION

Results of CGH in our series revealed only four tumours without CGH abnormalities, suggesting balanced or normal karyotypes. The findings in these cases are likely to be genuine rather than an artefact of contamination with normal cells because non-neoplastic tissue was not found in histological sections from three tumours, and accounted for only about 20% of tissue in one case. Gains and losses in the remaining cases were supported by the inclusion of controls in which we did not encounter the difficulties of interpretation of CG-rich regions presented in studies employing indirect labels (Kallioniemi et al, 1994; Reardon et al, 1997).

The findings of gain of 17q with reciprocal loss of 17p in 26% of our cases of medulloblastoma are consistent with cytogenetic reports (Biegel et al, 1989; Bigner et al, 1997), with two smaller series of cases studied using CGH (Schütz et al, 1996; Reardon et al, 1997), and with findings of LOH on 17p (Cogen et al, 1990; Blaeker et al, 1996). The relative importance of 17p loss and 17q gain remains unclear. In the search for potential tumour suppressor

 *© 1999 Cancer Research Campaign*

genes, reports of the potential role for TP53 have been inconsistent (Jaros et al, 1993; Batra et al, 1995; Phelan et al, 1995), but evidence for a medulloblastoma-related locus at 17p13.3, distal to TP53, has been presented (McDonald et al, 1994). In PNETs, where isochromosome formation may occur in the absence of other imbalances, as shown in cytogenetic studies (Biegel et al, 1989) and in two of our cases, it is possible that development of i(17q) is an early event. The importance of 17q gain is less clear, although our findings, particularly in terms of the correlation with GFAP immunoreactivity, suggest that 17q gain may have significance of its own. The associations found in this study between classic histology and imbalances of chromosome 17 have not been documented before in PNETs. Furthermore, the mutual exclusivity we demonstrated between tumours exhibiting gain of 17q and those showing immunoreactivity for GFAP has also not been described previously. This result contrasts with the results of one study in which the majority of PNETs with i(17q) showed ‘glial differentiation’ (Biegel et al, 1995). The GFAP gene is located at 17q21, but it is unclear how gain of 17q might interfere with the expression of this astroglial intermediate filament (Brenner et al, 1994).

Monosomy 22 has been documented in a wide range of CNS tumours (Heim and Mitelman, 1995) and is a feature of approximately 60% of CNS rhabdoid tumours, or atypical teratoid/rhabdoid tumours (Biegel et al, 1990; Rorke, 1997). It has been found in 0–30% of abnormal cases in cytogenetic reports of PNET, all cerebellar tumours (Biegel et al, 1989; Bhattachajee et al, 1997; Bigner et al, 1997), and results of microsatellite analyses have revealed LOH on 22q in similar proportions (Blaeker et al, 1996), but no correlations with clinical parameters have been made in any of these studies. Schütz and co-workers (1996) did not find any evidence of monosomy 22 in their CGH series and, although Reardon’s group (1997) found 5/27 cases with apparent monosomy 22, these were from a wide age range of patients with multiple CGH abnormalities. The tumours in our series with loss of 22 all came from particularly young patients, with a median age of 8 months at diagnosis. Loss of 22 was either an isolated imbalance or associated with only one other abnormality. These findings raise the question of the relationship of these four cases to CNS rhabdoid tumours, which characteristically show the varied immunoreactivities for neuroepithelial and non-neuroepithelial proteins and poor prognosis found in our cases. Though cytological pleomorphism is typically greater in CNS rhabdoid tumours than in medulloblastomas, the former may contain regions that resemble a classic medulloblastoma. For two of our cases with monosomy 22, tissue for histology was limited, and it is possible that insufficient sampling may have precluded a diagnosis of CNS rhabdoid tumour. However, retrospective review of the other two examples, where large portions of tumour had been submitted for histology, revealed no regions that might prompt a change in diagnosis.

Other imbalances detected in our series of medulloblastomas were broadly consistent with previous studies, but some require specific comments:

Loss of all or part of 9q, including 9q22, has been found in a proportion of PNETs in each of the published CGH series (Schütz et al, 1996; Reardon et al, 1997). Although all of our cases show loss of a region large enough to be seen clearly in a conventional cytogenetic preparation, interstitial losses of 9q have not been reported in cytogenetic studies of PNET. In contrast, LOH on 9q has been reported (Blaeker et al, 1996). Furthermore, 9q22 has been identified as the locus for the ‘*Drosophila* patched’ (*PTCH*) gene, mutated in sporadic medulloblastomas as well as in cases of Gorlin’s, or nevoid basal cell carcinoma syndrome, which predisposes to medulloblastoma (Vorechowsky et al, 1997; Wolter et al, 1997). One of our cases (case 24) with interstitial loss of 9q developed dermatological lesions characteristic of naevoid basal cell carcinoma syndrome after diagnosis of her medulloblastoma. Tumours with a nodular/desmoplastic architecture were significantly over-represented in our group with loss of 9q, corroborating a previously noted link between LOH on 9q and the desmoplastic variant of medulloblastoma (Schofield et al, 1995).

We found a high incidence of monosomy 8 (23%), only recorded in one previous cytogenetic study (Bhattachajee et al, 1997), in which the association with gain of 7 and i(17q) was also observed. Five of our medulloblastomas had gains including 12q23–q24. Although a finding not commented upon in their report, a similar number of cases in the series of Reardon et al (1997) also showed this imbalance. The epidermal growth factor receptor pathway substrate 8 (Eps8) gene maps to this region and overexpression of its product Eps8 enhances mitogenic response to epidermal growth factor (Fazioli et al, 1993; Wong et al, 1994). A role for Eps8 in tumour development or progression in these cases of PNET might therefore be postulated, particularly in view of recent attention given to the potential prognostic implications of EGFR expression (Gilbertson et al, 1997). Gain of 12q24 in our series was also associated with classic histology and absence of GFAP immunoreactivity.

Only 3/31 (10%) of the medulloblastomas in our study showed gene amplification, consistent with previous studies of primary tumours (Bigner and Vogelstein, 1990; Heim and Mitelman, 1993; Batra et al, 1994; Schütz et al, 1996; Reardon et al, 1997) and none of our cases had amplifications at previously reported loci. This gives added weight to the suggestion that the high proportion of cell lines with *MYC* amplification may be due to a selective advantage for in vitro growth conferred by this abnormality (Bigner and Vogelstein, 1990; Batra et al, 1994). Our results are at odds with the suggestion of Reardon et al (1997) that the combination of amplification and 9q deletion in medulloblastoma is analogous to *MYCN* amplification and 1p loss in neuroblastoma. While our cases with amplification did have 9q loss, they also had multiple additional abnormalities, and we had three cases of 9q loss that were not associated with gene amplification. Furthermore, in view of the rarity of gene amplification and variety of loci involved, it would seem unlikely that a single mechanism is at work, or that gene amplification plays an important role in the pathogenesis of medulloblastoma.

Conflicting results have emerged from studies of clinical prognostic indicators for PNET (Hubbard et al, 1989; Tait et al, 1990; Zerbini et al, 1993; Geyer et al, 1994; Bailey et al, 1995), but the presence of metastases at the time of diagnosis has featured consistently as an indicator of poor prognosis, and is supported by our findings. Prognosis has previously been linked to histological variant (Hubbard et al, 1989), with desmoplastic variants tending to have a more favourable outcome than classic medulloblastomas, but this has not been found to be the case in all reports (Choux et al, 1983) and, while our series showed a similar trend, it did not reach significance with the number of cases involved. GFAP immunoreactivity has been found to be predictive of a poor outcome (Janss et al, 1996) but a separate study (Maraziotis et al, 1992) found, like ours, that it had no independent prognostic significance. In the search for genetic abnormalities with potential prognostic value, previous attention has focused on aberrations of chromosome 17.

© 1999 Cancer Research Campaign

An association between LOH on 17p and adverse prognosis has been reported (Cogen et al, 1990; Batra et al, 1995), but these findings have not been reproduced in a separate series (Emadian et al, 1996) or in our CGH study. However, our findings in relation to loss of chromosome 22 and also to the expression of multiple proteins characteristic of different cell lineages in PNETs, suggest that they could be markers of a particularly poor prognosis.

Three out of four cases of cerebral PNET in our series demonstrated multiple chromosomal abnormalities, but patterns of chromosomal gains and losses that characterize medulloblastomas were not seen. There is little published cytogenetic or molecular genetic data for cerebral PNETs to allow comparison, but Burnett and coworkers (1997) found no cases of chromosome 17p loss in a series of eight supratentorial PNETs using molecular techniques, and suggest that supratentorial PNETs and medulloblastomas are genetically distinct. Loss of material from 3p was common to each of our abnormal cases which suggests the loss of a tumour suppressor gene from this region. The fragile histidine triad gene is a possible candidate, as it encompasses the fragile site at 3p14.2 and has been implicated in the development or progression of a variety of neoplasms, including renal cell carcinoma and small cell carcinoma of the lung (Sozzi et al, 1997; Xiao et al, 1997). The single case of cerebral PNET with amplified material (case 34) showed a similar pattern to that seen in a case of malignant glioma, with paired amplicons at 7p11.2p13 and 7q21.3q22 (Schröck et al, 1994).

In summary, we have demonstrated multiple chromosomal abnormalities in a series of PNETs using CGH and have found patterns of imbalance which are associated with tumour architecture and immunophenotype. We have also identified a subgroup of classic medulloblastomas that show similarities to CNS rhabdoid tumours and revealed patterns of abnormality in cerebral PNETs suggesting that supratentorial and posterior fossa PNETs may be genetically distinct.

### ACKNOWLEDGEMENTS

The CGH work (JCN) was funded by grants from the Wessex Cancer Trust and The Parthenon Trust.

### REFERENCES

Bailey CC, Gnekow A, Wellek S, Jones M, Round C, Brown J, Philips A and Neidhardt MK (1995) Prospective randomised trial of chemotherapy given before radiotherapy in childhood medulloblastoma. International Society of Paediatric Oncology (SIOP) and the (German) Society of Paediatric Oncology (GPO): SIOP II. *Med Pediatr Oncol* **25**: 166–178

Batra SK, Rasheed BKA, Bigner SH and Bigner DD (1994) Oncogenes and anti-oncogenes in human central nervous system tumours. *Lab Invest* **71**: 621–637

Batra SK, McLendon RE, Koo JS, Castelino-Prabhu S, Fuchs HE, Krischer JP, Friedman HS, Bigner DD and Bigner SH (1995) Prognostic implications of chromosome 17p deletions in human medulloblastomas. *J Neurooncol* **24**: 39–45

Bhattacharjee MB, Armstrong DD, Vogel H and Cooley LD (1997) Cytogenetic analysis of 120 primary brain tumours and literature review. *Cancer Genet Cytogenet* **97**: 39–53

Biegel JA, Rorke LB, Packer RJ, Sutton LN, Schut L, Bonner K and Emanuel BS (1989) Isochromosome 17q in primitive neuroectodermal tumours of the central nervous system. *Genes Chromosomes Cancer* **1**: 139–147

Biegel JA, Rorke LB, Packer RJ and Emanuel BS (1990) Monosomy 22 in rhabdoid or atypical tumours of the brain. *J Neurosurg* **73**: 710–714

Biegel JA, Rorke LB, Janss AJ, Sutton LN and Parmiter AH (1995) Isochromosome 17q demonstrated by interphase fluorescence in situ hybridization in primitive neuroectodermal tumours of the central nervous system. *Genes Chromosomes Cancer* **14**: 85–96

Bigner SH and Vogelstein B (1990) Cytogenetics and molecular genetics of malignant gliomas and medulloblastoma. *Brain Pathol* **1**: 12–18

Bigner SH, McLendon RE, Fuchs H, McKeever PE and Friedman HS (1997) Chromosomal characteristics of childhood brain tumours. *Cancer Genet Cytogenet* **97**: 125–134

Blaeker H, Rasheed BKA, McLendon RE, Friedman HS, Batra SK, Fuchs HE and Bigner SH (1996) Microsatellite analysis of childhood brain tumours. *Genes Chromosomes Cancer* **15**: 54–63

Brenner M (1994) Structure and transcriptional regulation of the GFAP gene. *Brain Pathol* **4**: 245–257

Burnett ME, White EC, Sih S, von Haken MS and Cogen PH (1997) Chromosome arm 17p deletion analysis reveals molecular genetic heterogeneity in supratentorial and infratentorial primitive neuroectodermal tumours of the central nervous system. *Cancer Genet Cytogenet* **97**: 25–31

Choux M, Lena G and Hassoun J (1983) Prognosis and long-term follow-up in patients with medulloblastoma. *Clin Neurosurg* **30**: 246–277

Cogen PH, Daneshvar L, Metzger AK and Edwards MSB (1990) Deletion mapping of the medulloblastoma locus on chromosome 17p. *Genomics* **8**: 279–285

Ellison DW, Steart PV, Gatter KC and Weller RO (1995) Apoptosis in cerebral astrocytic tumours and its relationship to expression of the Bcl-2 and P53 proteins. *Neuropathol Appl Neurobiol* **21**: 352–361

Ellison DW and Love S (Eds) (1998) Embryonal neuroepithelial neoplasms of the CNS. In: *Neuropathology.* Mosby: London.

Emadian SM, McDonald JD, Gerken SC and Fults D (1996) Correlation of chromosome 17p loss with clinical outcome in medulloblastoma. *Clin Cancer Res* **2**: 1559–1564

Fazioli F, Minichiello L, Matoska V, Castagnino, Miki T, Wong WT and Di Fiore PP (1993) Eps8, a substrate for the epidermal growth factor receptor kinase, enhances EGF-dependent mitogenic signals. *EMBO J* **12**: 3799–3508

Geyer JR, Zeltzer PM, Boyett JM, Rorke LB, Stanley P, Albright L, Wisoff JH, Milstein JM, Allen JC, Finlay JL, Ayers GD, Shurin SB, Stevens KR and Bleyer WA (1994) Survival of infants with primitive neuroectodermal tumours or malignant ependymomas of the CNS treated with eight drugs in 1 day: a report from the children's cancer group. *J Clin Oncol* **12**: 1607–1615

Giangaspero F, Chieco P, Ceccarelli C, Lisignoli G, Pozzuoli R, Gambacorta M, Rossi G and Burger PC (1991) 'Desmoplastic' versus 'classic' medulloblastoma: comparison of DNA content, histopathology and differentiation. *Virchows Archiv A Pathol Anat* **418**: 207–214

Gilbertson RJ, Perry RH, Kelly PJ, Pearson ADJ and Lunec J (1997) Prognostic significance of HER2 and HER4 coexpression in childhood medulloblastoma. *Cancer Res* **57**: 3272–3280

Heim S and Mitelman F (1995) Tumours of the nervous system. In: *Cancer Cytogenetics; Chromosomal and Molecular Genetic Aberrations of Tumour Cells*, 2nd edn, pp. 432–454. Wiley-Liss: New York

Hubbard JL, Scheithauer BW, Kispert DB, Carpenter SM, Wick MR and Laws ER Jr (1989) Adult cerebellar medulloblastomas: the pathological, radiographic and clinical disease spectrum. *J Neurosurg* **70**: 536–544

ISCN (1995) *An International System for Human Cytogenetic Nomenclature*, Mitelman F (ed). S Karger: Basel

Janss AJ, Yachnis AT, Silber JH, Trojanowski JQ, Lee VM-Y, Sutton LN, Perilongo G, Rorke LB and Phillips PC (1996) Glial differentiation predicts poor clinical outcome in primitive neuroectodermal brain tumours. *Ann Neurol* **39**: 481–489

Jaros E, Lunec J, Perry RH, Kelly PJ and Pearson ADJ (1993) p53 overexpression identifies a group of central primitive neuroectodermal tumours with a poor prognosis. *Br J Cancer* **68**: 801–807

Kallioniemi O-P, Kallioniemi A, Piper J, Isola J, Waldman FM, Gray JW and Pinkel D (1994) Optimising comparative genomic hybridization for analysis of DNA sequence copy number changes in solid tumours. *Genes Chromosomes Cancer* **10**: 231–243

Kleihues P, Burger PC and Scheithauer BW (1993) *Histological Typing of Tumours of the Central Nervous System*. Springer: Berlin

Maraziotis T, Perentes E, Karamitopoulo E, Nakagawa Y, Gessaga EC, Probst A and Frankfurter A (1992) Neuron-associated class III b-tubulin isotype, retinal S-antigen, synaptophysin, and glial fibrillary acidic protein in human medulloblastomas: a clinicopathological analysis of 36 cases. *Acta Neuropathol* **84**: 355–363

McDonald JD, Daneshvar L, Willert JR, Matsumura K, Waldman F and Cogen PH (1994) Physical mapping of chromosome 17p13.3 in the region of a putative tumour suppressor gene important in medulloblastoma. *Genomics* **23**: 229–232

Mertens F, Johansson B and Mitelman F (1994) Isochromosomes in neoplasia. *Genes Chromosomes Cancer* **10**: 221–230

Miller SA, Dykes DD and Polesky HF (1988) A simple salting out procedure for extracting DNA from human nucleated cells. *Nucleic Acids Res* **16**: 1215–1215

© 1999 Cancer Research Campaign

Packer RJ (1995) Brain tumours in children. *Curr Opin Ped* **7**: 64–72
Phelan CM, Liu L, Ruttledge MH, Müntzning K, Ridderheim P-A and Collins VP (1995) Chromosome 17 abnormalities and lack of TP53 mutations in paediatric central nervous system tumours. *Hum Genet* **96**: 684–690
Pinkel D, Landegent J, Collins C, Fuscoe J, Segraves R, Lucas J and Gray J (1988) Fluorescence in situ hybridization with human chromosome-specific libraries: detection of trisomy 21 and translocations of chromosome 4. *Proc Natl Acad Sci USA* **85**: 9138–9142
Reardon DA, Michalkiewicz, Boyett JM, Sublett JE, Entrekin RE, Ragsdale ST, Valentine MB, Behm FG, Li H, Heideman RL, Kun LE, Shapiro DN and Look AT (1997) Extensive genomic abnormalities in childhood medulloblastoma by comparative genomic hybridization. *Cancer Res* **57**: 4042–4047
Rorke LB (1997) Atypical teratoid/rhabdoid tumours. In *Tumours of the Nervous System*, Kleihues P and Cavanee WK (eds), pp 110–111. International Agency for Research on Cancer: Lyon
Schofield D, West DC, Anthony DC, Marshal R and Sklar J (1995) Correlation of loss of heterozygosity at chromosome 9q with histological sub-type in medulloblastomas. *Am J Pathol* **146 (2)**: 472–480
Schröck E, Thiel G, Lözanova T, du Manoir S, Meffert M-C, Jauch A, Speicher MR, Nürnberg P, Vogel S, Jänisch W, Donis-Keller H, Ried T, Witkowski R and Cremer T (1994) Comparative genomic hybridization of human malignant gliomas reveals multiple amplification sites and nonrandom chromosomal gains and losses. *Am J Pathol* **144**: 1203–1218
Schütz BR, Scheurlen W, Krauss J, du Manoir S, Joos S, Bentz M and Lichter P (1996) Mapping of chromosomal gains and losses in primitive neuroectodermal tumours by comparative genomic hybridization. *Genes Chromosomes Cancer* **16**: 196–203
Sozzi G, Tornielli S, Tagliabue E, Sard L, Pezella F, Pastorino U, Minoletti F, Pilooti S, Ratcliffe C, Veronese ML, Goldstraw P, Huebner K, Croce CM and Pierotti MA (1997) Absence of Fhit protein in primary lung tumours and cell lines with FHIT gene abnormalities. *Cancer Res* **57**: 5207–5212
Tait DM, Thornton-Jones H, Bloom HJG, Lemerle J and Morris-Jones P (1990) Adjuvant chemotherapy for medulloblastoma: the first multi-centre control trial of the International Society of Paediatric Oncology (SIOP I). *Eur J Cancer* **26**: 464–469
Tomlinson FH, Scheithauer BW and Jenkins RB (1992) Medulloblastoma. II: a pathobiologic overview. *J Child Neurol* **7**: 240–252
Vorechovsky I, Tingby O, Hartman M, Strömberg B, Nister M, Collins VP and Toftgärd R (1997) Somatic mutations in the human homologue of *Drosophila* patched in primitive neuroectodermal tumours. *Oncogene* **15**: 361–366
Wolter M, Reifenberger J, Sommer C, Ruzicka T and Reifenberger G (1997) Mutations in the human homologue of the *Drosophila* segment polarity gene *patched (PTCH)* in sporadic basal cell carcinomas of the skin and primitive neuroectodermal tumours of the central nervous system. *Cancer Res* **57**: 2581–2585
Wong WT, Carlomagno F, Druck T, Barletta C, Croce CM, Huebner K, Kraus MH and Di Fiore PP (1994) Evolutionary conservation of the EPS8 gene and its mapping to human chromosome 12q23–q24. *Oncogene* **9**: 3057–3061
Xiao GH, Jin F, Klein-Szanto AJ, Goodrow TL, Linehan MW and Yeung RS (1997) The FHIT gene product is highly expressed in the cytoplasm of renal tumular epithelium and is down-regulated in kidney cancers. *Am J Pathol* **151**: 1541–1547
Zerbini C, Gelber RD, Weinberg D, Sallan SE, Barnes P, Kupsky W, Scott RM and Tarbell NJ (1993) Prognostic factors in medulloblastoma including DNA ploidy. *J Clin Oncol* **11**: 616–622

*© 1999 Cancer Research Campaign*
